# Risk factors associated with extracranial atherosclerosis in old patients with acute ischemic stroke

**DOI:** 10.1038/s41598-018-31026-z

**Published:** 2018-08-22

**Authors:** Lili Wang, Maolin He, Yun Zhang

**Affiliations:** Department of Neurology, Beijing Shijitan Hospital, Capital Medical University, Beijing, China

## Abstract

The aim of this study was to identify risk factors associated with extracranial atherosclerosis (ECAS) in old Chinese patients with ischemic stroke. Eligible patients were divided into two groups: <60years group and ≥60 years group. The incidence, severity of ECAS and risk factors related to ECAS were compared between the two groups. In total, 921 patients were included in the analysis. The distribution of ECAS between the two age groups did not show difference (P = 0.747).But severe ECAS stenosis was more common in <60years group than in ≥60years group (*x*^2^ = 8.307, P = 0.013). Compared with <60years group, factors contributed to higher risk of ECAS in ≥60year group were hypertension(OR = 6.279, *P* = 0.000), heart disease(OR = 5.618, *P* = 0.032) and atrial fibrillation(OR = 7.477, *P* = 0.015). However, though smoking was higher in the <60years group (*x*^2^ = 7.664, *P* = 0.010) than in ≥60year group, multivariate analysis showed the difference was not significant(*P* = 0.879). Further studies should focus on risk factors in young ECAS patients. Different risk factors might contribute to ECAS in old age groups compared to young groups. Risk factor prevention/control measures should be strengthened in those with high risk of ECAS for decreasing stroke risk.

## Introduction

Large-artery atherosclerosis is a common cause of ischemic stroke. Although intracranial atherosclerosis (ICAS) is the major vascular lesions in patients with ischemic stroke in Asian, extracranial atherosclerosis (ECAS) also contributed to mortality or morbidity in 20% to 30% ischemic stroke patients in Asian^1,2^. Previous studies have identified several risk factors,including older age, male gender, smoke, and hyperlipidemia, contributed to ECAS, but the results are controversial^3–5^. Several studies have shown that older age increased risk for ECAS and concurrence extracranial and intracranial atherosclerosis (EICAS) in China^3^. However, others have reported that older Chinese patients with atherosclerotic stenosis tend to have more ICAS^5^. Furthermore, a meta-analysis in Asian population found that age did not show significant difference between ICAS and ECAS^4^. Till now, risk factors associated with ECAS in different age groups have not been reported.

Above all, age difference in the distribution of ECAS has not been fully discussed before in Chinese patients with ischemic stroke. The purpose of this study was to elucidate the relationship of age with ECAS and to investigate the difference in the risk factors associated with ECAS between different age groups.

## Results

### Patient Characteristics in ECAS and other three groups

From January 2010 to December 2012, a total of 921 patients with acute ischemic stroke (within 7 days after onset) were enrolled in the present study. There were 367(39.8%) female patients, with a median age of 66.74 years. All patients were divided into 4 groups based on stenosis subtypes: ECAS, 140(15.2%); ICAS, 280 (30.4%); EICAS, 160(17.4%); none cranial atherosclerosis (NCAS), which had no cranial atherosclerosis, 341 (37.0%).The clinical characteristics of the four groups were shown in Table 1. Risk factors related to ECAS compared with NCAS or ICAS or EICAS were also showed in Table 1.

**Table 1:** The differences in the characteristics between ECAS and other three groups

	NCAS (n = 341)	ECAS (n = 140)	ICAS (n = 280)	IECAS (n = 160)	P_1_	P_2_	P_3_	P_4_
Age (<60year)	125 (36.7%)	42 (30.0%)	85 (30.4%)	35 (21.9%)	0.172	1.000	0.114	0.010
Diabetes mellitus	45 (13.2%)	25 (17.9%)	72 (25.7%)	81 (50.16%)	0.201	0.085	0.000	0.000
Hypertension	147 (43.1%)	77 (55.0%)	160 (57.1%)	103 (64.4%)	0.021	0.754	0.124	0.000
TC	56 (16.4%)	19 (13.6%)	72 (25.7%)	25 (15.6%)	0.491	0.005	0.628	0.003
TG	92 (27.0%)	31 (22.1%)	85 (30.4%)	56 (35.0%)	0.301	0.083	0.016	0.075
HDL	53 (15.5%)	115 (82.1%)	242 (86.4%)	138 (86.3%)	0.000	0.310	0.344	0.000
LDL	64 (18.8%)	30 (21.4%)	76 (27.1%)	41 (25.6%)	0.528	0.234	0.417	0.072
History of stroke	46 (13.6%)	31 (22.1%)	40 (14.3%)	40 (25.0%)	0.028	0.053	0.588	0.003
Heart disease	43 (12.6%)	41 (29.3%)	87 (31.1%)	59 (36.9%)	0.000	0.737	0.178	0.000
Family history of stroke	13 (3.8%)	13 (9.3%)	10 (3.6%)	13 (8.1%)	0.024	0.022	0.838	0.017
Smoke	97 (28.7%)	35 (25.0%)	78 (27.9%)	46 (28.7%)	0.433	0.561	0.515	0.860
Sex (female)	141 (41.3%)	57 (40.7%)	109 (38.9%)	60 (37.5%)	0.943	0.751	0.635	0.843
Atrial fibrillation	17 (5.0%)	33 (23.6%)	18 (6.4%)	39 (24.4%)	0.000	0.000	0.893	0.000

P_1_: compared between ECAS and NCAS.

P_2_: compared between ECAS and ICAS.

P_3_: compared between ECAS and EICAS.

P_4_: compared among four groups.

TG: ≥1.7 mmol/L;TC: ≥5.7 mmol/L; HDL: ≤0.91 mmol/L;LDL: ≥3.6 mmol/L.

NCAS: none cranial atherosclerosis.

ECAS:extracranial atherosclerosis.

ICAS: intracranial atherosclerosis.

EICAS: concurrence extracranial and intracranial atherosclerosis.

### ECAS distribution in two age groups

Distribution of ECAS,ICAS between ≥60 and <60 years group (ECAS15.5% versus 14.6%,P = 0.747; ICAS30.8% versus 29.6%,P = 0.757) did not show significant difference. The stenosis degree of ECAS and ICAS was significantly different between <60years group and ≥60years group. Otherwise, the distribution of EICAS between ≥60 and <60 years group had significant difference, the stenosis degree of EICAS between the two groups had no significant difference.Distribution of cerebral artherosclerosis in different age groups was show in Table 2. Baseline characteristics of different age groups were show in Table 3.

**Table 2:** Age with position and severity of cranial artery stenosis (CAS.

	<60years (n = 287)	≥60years (n = 634)	P value
Distribution of CAS			
NCAS	125 (43.6%)	216 (34.1%)	0.010*
ICAS	85 (29.6%)	195 (30.8%)	0.757#
ECAS	42 (14.6%)	98 (15.5%)	0.747&
IECAS	35 (12.2%)	125 (19.7%)	0.006**
Severity of ICAS			
50−69%	61 (71.8%)	167 (85.6%)	0.013
70–99%	14 (16.5%)	20 (10.3%)	
100%	10 (11.8%)	8 (4.1%)	
Severity of ECAS			
50–69%	32 (76.2%)	91 (92.9%)	0.013
70–99%	7 (16.7%)	6 (6.1%)	
100%	3 (7.1%)	1 (1.0%)	
Severity of EICAS			
50−69%	23 (65.7%)	85 (68.0%)	0.955
70–99%	8 (22.9%)	28 (22.4%)	
100%	4 (11.4%)	12 (9.6%)	

*Compared among four groups.

^#^Compared between ECAS and other three groups(EICAS,ICAS and NCAS).

^&^Compared between ICAS and other three groups(EICAS,ECAS and NCAS).

^**^Compared between EICAS and other three groups(ICAS,ECAS and NCAS).

**Table 3:** Baseline characteristics by age.

	<60years (n = 287)	≥60years (n = 634)	P_1_ value	P_2_ value	OR(95%CI)
Diabetes mellitus	51 (17.8%)	172 (27.1%)	0.002	0.011	1.633 (1.119–2.383)
Hypertension	120 (41.8%)	367 (57.9%)	0.000	0.000	1.827 (1.340–2.491)
TC	52 (18.1%)	120 (18.9%)	0.785		
TG	93 (32.4%))	171 (27.0%)	0.099		
HDL	164 (57.1%)	384 (60.6%)	0.346		
LDL	66 (23.0%)	145 (22.9%)	1.000		
History of stroke	37 (13.0%)	120 (16.5%)	0.029	0.140	1.392 (0.898–2.158)
Heart disease	51 (17.8%)	179 (28.2%)	0.001	0.018	1.628 (1.086–2.441)
Family history of stroke	27 (9.4%)	22 (3.5%)	0.000	0.000	0.218 (0.103–0.462)
Sex (female)	79 (27.5%)	288 (45.4%)	0.000		
Smoke	121 (42.2%)	135 (21.4%)	0.000	0.000	0.370 (0.267–0.513)
Atrial fibrillation	20 (7.0%)	87 (13.7%)	0.004	0.005	2.586(1.325–5.405)

P_1_ value: compared between <60years group and ≥60years group (univariate analysis).

P_2_ value: compared between <60years group and ≥60years group (multivariate analysis).

### Risk factors with ECAS in two age groups

The difference in baseline characteristics for ECAS in two age groups is shown in Table 4.

**Table 4:** Risk factors related to ECAS in different age groups.

	ECAS		P_1_	P_2_	OR (95%CI)
	<60years (n = 42)	≥60years (n = 98)			
Sex (woman)	16 (38.1%)	41 (41.8%)	0.711		
Diabetes mellitus	7 (16.7%)	18 (18.4%)	1.000		
Hypertension	11 (26.2%)	66 (67.3%)	0.000	0.000	6.279 (2.586–15.246)
TC	7 (16.7%)	12 (12.2%)	0.591		
TG	11 (26.2%)	20 (20.4%)	0.507		
HDL	36 (85.7%)	79 (80.6%)	0.631		
LDL	13 (31.0%)	17 (17.3%)	0.114		
History of stroke	5 (11.9%)	26 (26.5%)	0.075		
Heart disease	2 (4.8%)	39 (39.8%)	0.002	0.032	5.618 (1.160–27.198)
Family history of stroke	4 (9.5%)	9 (9.2%)	1.000		
Smoke	17 (40.5%)	18 (18.4%)	0.01	0.879	1.083 (0.388–3.017)
Atrial fibrillation	2 (4.8%)	31 (31.6%)	0.001	0.015	7.477 (1.474–37.914)

P_1_ value: compared between <60years group and ≥60years group (univariate analysis).

P_2_ value: compared between <60years group and ≥60years group (multivariate analysis).

TG: ≥1.7 mmol/L;TC: ≥5.7 mmol/L; HDL: ≤0.91 mmol/L;LDL: ≥3.6 mmol/L.

ECAS:extracranial atherosclerosis.

Compared to <60 years group, patients in ≥60 years group were with higher proportion of hypertension,heart disease and atrial fibrillation.

## Discussion

In this study, we investigated the relationship of age with ECAS and identified risk factors associated with ECAS in different age groups(<60 and ≥60 years group). Our results showed that distribution of ECAS has no age variation in China. Patients with 100% ECAS was approximately 7.10-fold more frequent in <60 years group than that in ≥60 years group. Patients with ECAS in <60 years group smoked more cigarette, which help to explain the difference. However, there was significant difference in the distribution of EICAS between the two age groups. but stenosis degree of EICAS between the two groups did not show significant difference.

Previous studies have documented age variations in the distribution of ECAS. But the results were inconsistent. Many studies have shown that ECAS distributed more in older ages than in young ages^1,3,6,7^. Others have reported that older Chinese patients with atherosclerotic stenosis tend to have more ICAS than ECAS^5^. Furthermore, a meta-analysis in Asian population found that age did not show significant difference between ICAS and ECAS^4^. In this study, our results confirmed that there was no age difference between ICAS and ECAS.

Male, smoke and hyperlipidemia was also identified as predictors for ECAS in ischemic stroke in previous studies^8–12^. Diabetes mellitus, history of stroke and heart disease were associated with increased risk of ECAS in ischemic stroke^7,13–15^. However, our study did not show significant difference of sex, smoke and diabetes mellitus between ECAS and NCAS. Other risk factors were consistent with previous studies. But our data showed family history of stroke, atrial fibrillation and lower TC was more often in ECAS than in ICAS. Diabetes mellitus and TG difference between ECAS and EICAS was also significant.

The difference of risk factors for ECAS in different age groups has not been reported. First, we investigated baseline characteristic difference in two age groups divided by age 60. We found that hypertension, diabetes mellitus, heart disease, atrial fibrillation and female significantly prevalent in old patients. We also found older patients consumed less cigarettes. Second, we further investigated risk factors for ECAS in two age groups. The results showed positive correlation between hypertension and heart disease with ECAS in ≥60 years group. Smoke was more common in <60years group than in ≥60 years group,but the difference was not significant^9,16^.

The results of our study improve the understanding of the incidence, severity of ECAS and risk factors related to ECAS in old patients of China. ECAS is one of the most common underlying cause for early recurrent stroke^17,18^. Primary prevention,including lifestyle improve and risk factors control should be strengthened to reduce the incidence of ECAS in the China, especial in older persons.

There were limitations of our study. First, it was done in one hospital, so it may not accurately reflect what happens in the general population. Second, we only examined the common baseline variables, other related risk factors needs to be further validated.

In conclusion, there exists no age difference in the distribution of symptomatic ECAS in China. But the proportion of severe ECAS is higher in the young age than that in old age. Difference in risk factors helps to explain the variation in ECAS prevalence. To decrease the risk of stroke, the government should support early cerebrovascular examination in high-risk populations. Risk factor prevention/control measures should be strengthened in those with high risk of ECAS.

## Methods

### Patients

This study was conducted using prospective data from Neurology Department of Beijing Shijitan Hospital. In the present study, inclusion criteria included patients with acute ischemic stroke (transient ischemic attack, cerebral thrombosis, within 7 days after onset). There were 921 patients who were eligible for this study, including 140 patients with ECAS, 160 patients with EICAS, 280patients with ICAS, and 341 patients with NCAS. We excluded patients who had other obvious etiologies such as arterial dissection, Moyamoya disease, vasculitis. The study was approved by the hospital ethics committee on clinical research. All patients provided written informed consent for participation. All methods were performed in accordance with the relevant guidelines and regulations.

### Cerebral Atherosclerotic Stenosis

All patients underwent conventional magnetic resonance imaging (MRI). Intracranial vessels were judged by transcranial Doppler (TCD). Extracranial vessels were examined by duplex color Doppler ultrasound. Cranial artherosclerosis was further evaluated and confirmed by other vascular imaging methods including magnetic resonance angiography (MRA), computed tomography angiography (CTA) or digital subtraction angiography (DSA)^6,19^.

Intracranial arteries were evaluated in the intracranial segment of the internal carotid artery (ICA) and vertebral arteries (VA), the basilar artery (BA), the middle cerebral artery (M1), the anterior cerebral artery (A1), and the posterior cerebral artery (P1). The extracranial arteries were evaluated in the proximal ICA and proximal VA. More than 50% atherosclerotic stenosis or occlusion of the large arteries was considered significant stenosis and included in this study. ICAS was defined as ≥50% diameter reduction on TCD. ECAS was defined as ≥50% diameter reduction on Carotid duplex^2,20^. ICAS was further confirmed as ≥50% diameter reduction on magnetic resonance angiography.ECAS was defined as ≥50% diameter reduction on Carotid duplex. All patients with excranial artherosclerosis was further evaluated and confirmed by other vascular imaging methods including contrast-enhanced MRA, CTA or DSA.None cranial atherosclerosis (NCAS) was that who had no cranial atherosclerosis. NCAS was first judged by TCD and carotid duplex. NACS were further confirmed by MRA or CTA.

According to the presence and location of cerebral atherosclerotic stenosis, patients were classified into 4 groups: NCAS group, ICAS group, ECAS group and EICAS group. According to the severity of stenosis, the patients with cranial artery stenosis were classified into 3 groups: 50% to 69% stenosis, 70% to 99% and 100% stenosis.

### Risk Factors

We collected baseline information at admission including age, gender, hypertension, diabetes mellitus, hyperlipidemia, smoking, history of stroke, heart disease, atrial fibrillation and family history of stroke. Hypertension was defined as regular treatment with antihypertensive agent before admission or diagnosed at discharge. Diabetes mellitus was defined as the use of antidiabetic medication or diagnosed at discharge. Current smoker was defined as smoking continuously ≥1 cigarette a day for at least 1 year. History of stroke was defined as the experience of ischemic stroke and transient ischemic attack. Hyperlipidemia was defined as low-density lipoprotein cholesterol (LDL) ≥3.6 mmol/L, or high-density lipoprotein cholesterol (HDL) ≤0.91 mmol/L, or total cholesterol (TC) ≥5.7 mmol/L or triglyceride (TG) ≥1.7 mmol/L at the time of admission. Heart disease was defined as a known history of acute coronary syndrome or chronic coronary artery disease. Family history of stroke was defined as the stroke experience in the parents and siblings of the enrolled patients.

### Statistical Analysis

The χ2 test was used to compare categorical variables and independent samples *t* test was used to compare continuous variables. Multivariable logistic regression was further performed to determine the relationship of age with ECAS and to identify independent predictors of ECAS in different age groups. all statistical analysis was performed with SPSS software version22 (IBM, New York) . All tests were 2-sided, *P* < 0.05 was considered statistically significant.

### Ethics approval and consent to participate

The study protocols were approved by the ethics committees of Beijing Shijitan Hospital, Capital Medical University. Written informed consent was obtained from all patients participating in the study.

## Data Availability

The data in this study are available from the corresponding author on reasonable request.
